# Neural progenitor cell proliferation in the hypothalamus is involved in acquired heat tolerance in long-term heat-acclimated rats

**DOI:** 10.1371/journal.pone.0178787

**Published:** 2017-06-19

**Authors:** Kentaro Matsuzaki, Masanori Katakura, Naotoshi Sugimoto, Toshiko Hara, Michio Hashimoto, Osamu Shido

**Affiliations:** 1 Department of Environmental Physiology, Faculty of Medicine, Shimane University, Izumo, Japan; 2 Department of Nutritional Physiology, Faculty of Pharmaceutical Sciences, Josai University, Sakado, Saitama, Japan; 3 Department of Physiology, Graduate School of Medical Science, Kanazawa University, Kanazawa, Japan; St. Joseph's Hospital and Medical Center, UNITED STATES

## Abstract

Constant exposure to moderate heat facilitates progenitor cell proliferation and neuronal differentiation in the hypothalamus of heat-acclimated (HA) rats. In this study, we investigated neural phenotype and responsiveness to heat in HA rats’ hypothalamic newborn cells. Additionally, the effect of hypothalamic neurogenesis on heat acclimation in rats was evaluated. Male Wistar rats (5 weeks old) were housed at an ambient temperature (T_a_) of 32°C for 6 days (STHA) or 40 days (LTHA), while control (CN) rats were kept at a T_a_ of 24°C for 6 days (STCN) or 40 days (LTCN). Bromodeoxyuridine (BrdU) was intraperitoneally injected daily for five consecutive days (50 mg/kg/day) after commencing heat exposure. The number of hypothalamic BrdU-immunopositive (BrdU+) cells in STHA and LTHA rats was determined immunohistochemically in brain samples and found to be significantly greater than those in respective CN groups. In LTHA rats, approximately 32.6% of BrdU+ cells in the preoptic area (POA) of the anterior hypothalamus were stained by GAD67, a GABAergic neuron marker, and 15.2% of BrdU+ cells were stained by the glutamate transporter, a glutamatergic neuron marker. In addition, 63.2% of BrdU+ cells in the POA were immunolabeled with c-Fos. Intracerebral administration of the mitosis inhibitor, cytosine arabinoside (AraC), interfered with the proliferation of neural progenitor cells and acquired heat tolerance in LTHA rats, whereas the selected ambient temperature was not changed. These results demonstrate that heat exposure generates heat responsive neurons in the POA, suggesting a pivotal role in autonomic thermoregulation in long-term heat-acclimated rats.

## Introduction

The precise maintenance of core body temperature is a fundamental homeostatic function governed by the central nervous system in humans and rodents. Previous studies have shown preoptic area (POA) of the hypothalamus as the dominant thermoregulatory region, and other hypothalamic areas are also involved in maintaining afferent and efferent neuronal pathways contributing to monitoring core and skin temperatures and controlling peripheral thermo-effectors [1–6]. Recently, neuronal circuits, as well as neural transmissions, involved in temperature regulation have been studied. For example, in response to heat stress, warmth receptors within the dorsal root ganglion provide excitatory input to POA neurons via relay neurons in the dorsal lateral parabrachial nucleus [4, 7, 8]. Activation of glutamatergic neurons in various POA subareas results in severe hypothermia, whereas heat-activated GABAergic neurons in the POA reduce the activity of cold-activated neurons in the dorsomedial hypothalamic nucleus (DMH), which increase thermogenesis and physical activity [9]. Therefore, glutamatergic and/or GABAergic neurons of the POA in the hypothalamus play an important role in maintaining a stable core temperature via afferent inputs from thermoreceptors. Conversely, it has been shown that neural progenitor cells are present in the ependymal layer of the third ventricle [10–13]. In response to varied stimuli, hypothalamic progenitor cells proliferate and newborn cells migrate into the hypothalamic parenchyma where they differentiate into neuronal or glial cells [10]. The newborn hypothalamic neurons can be functionally and morphologically integrated into neuronal circuits by forming synapses where they may have functional roles in, for instance, a feeding and metabolic control system in rodents [14, 15]. Thus, neurogenesis and related remodeling of various neuronal networks in the hypothalamic area may play an important role in the regulation of hypothalamic function.

In humans and rodents, passive exposure to moderate heat is known to induce heat acclimation, which increases endurance for acute heat stress [16, 17]. A large number of studies reported that numerous physiological changes are confirmed in heat acclimated (HA) animals, e.g., enhanced sweating and cutaneous vasodilation, an increase in plasma volume, a decrease in core temperature at rest or during exercise, and reduced heart rate [16, 17]. The process of heat acclimation is classified into two types, namely, short-term HA (STHA) and long-term HA (LTHA), depending on the length of the term of heat stimuli [18]. Thermoregulatory changes of STHA can be organized within a few days (approximately 5 days) of heat exposure and are rapidly lost after the end of the thermal exposure, while those of LTHA are established around 4–5 weeks and are stable and sustained [19, 20]. In LTHA, changes in the ratio of thermosensitive to insensitive POA neurons after the improvement of HA rats ascertain the existence of neuronal plasticity in the hypothalamic regions responsible for thermoregulatory integration during heat acclimation [21]. Furthermore, several studies performed from various points of view, e.g., gene expression and morphological changes, have been reported in the synaptic structures in POA of HA rats [17, 22]. Thus, sustained functional and/or morphological changes of POA in the hypothalamus may be anticipated in LTHA rats. We have previously reported that heat exposure generates hypothalamic neurons in HA rats, and a part of newborn neurons are then integrated into hypothalamic neuronal circuits [23, 24]. We demonstrated that hypothalamic progenitor cell proliferation is promoted within a few days from the onset of heat exposure, and newborn cells differentiate into neurons around 4–5 weeks after commencing heat exposure. We also found that heat exposure-induced neurogenesis and acquired heat tolerance decline in aged rats [25]. These observations indicate that heat exposure facilitates proliferation of neuronal progenitor cells in the hypothalamus and promote differentiation into neurons, which may have a certain relationship for establishing LTHA in rats.

The present study aimed to clarify the involvement of hypothalamic newborn neurons in thermoregulatory responses in HA rats. To confirm the effect of progenitor cell proliferation and neural differentiation by heat exposure, the expression levels of cell proliferation and neural differentiation markers in the hypothalamus of HA rats were analyzed. Other related aspects such as (1) expression levels of GABAergic and/or glutamatergic neuron markers in hypothalamic newborn cells of HA rats were assessed, 2) identify hypothalamic newborn cells that expressed c-Fos protein, a marker for neuronal activation, to identify neurons that respond to heat stimuli, and 3) determine whether inhibition of heat exposure-induced hypothalamic neurogenesis by the mitosis inhibitor, cytosine arabinoside (AraC), affects acquired heat tolerance and behavioral thermoregulation in LTHA rats.

## Materials and methods

### Ethics statement

All animal experiments were performed adopting the Guidelines for Animal Experimentation of the Shimane University Faculty of Medicine, compiled from the Guidelines for Animal Experimentation of the Japanese Association for Laboratory Animal Science. The protocol in this study was approved by the Committee on the Ethics of Animal Experiments of the Shimane University.

### Experimental schedule

Male Wistar rats (5 weeks old) were obtained from Japan SLC Inc. (Shizuoka, Japan). Rats were housed in transparent plastic cages with wood chippings and initially maintained at an ambient temperature (T_a_) of 24.0 ± 0.1°C, with a relative humidity of 50 ± 5% under a 12:12-h light–dark cycle. At first, rats were anesthetized and a temperature transmitter (TA10TA-F40; Data Sciences International, St Paul, MN, USA) was implanted in their intraperitoneal cavity. Rats were allowed to recover from surgery for at least 10 days prior to data collection. All rats were housed individually to avoid crosstalk of radio telemetry. After the recovery period, the rats for heat acclimation (HA) were subjected to a constant T_a_ of 32.0 ± 0.2°C for 6 days (short-term heat-acclimated rats; STHA rats) or 40 days (long-term heat-acclimated rats; LTHA rats), with a relative humidity of 40 ± 10%, whereas each group of control rats (STCN and LTCN) was continuously kept at 24.0 ± 0.1°C. To detect newly generated cells, bromodeoxyuridine (BrdU; Sigma, St Louis, MO, USA) was dissolved in saline (10 mg/mL) and injected daily into the abdominal cavity of rats (50 mg/kg/day) for 5 consecutive days after starting heat exposure. On the 6^th^ or 40^th^ day (depending on the group) after the start of the heat exposure period, all rats were loaded to a T_a_ of 36°C to upregulate the expression of the c-Fos protein. Brain samples were removed and used for immunohistochemical analysis and Western blotting.

### Immunohistochemistry

Rats were anesthetized and transcardially perfused with ice cold 10N Mildform (Wako Pure Chemical Industries Ltd, Japan), followed by a saline perfusion. Brains were removed, fixed overnight at 4°C in 10N Mildform, and immersed in a 20% (w/v) sucrose solution. A cryostat was used to prepare brain sections (40-μm thickness), which were collected as free-floating sections. For the detection of BrdU incorporation, brain sections were incubated in 50% formamide/2×standard sodium citrate for 2 h at 65°C, incubated in 2 N HCl for 30 min at 37°C, rinsed in 100 mM boric acid (pH 8.5) for 10 min at 25°C, and washed with 0.25% Triton X-100 in Tris-buffered saline (TBS; pH 7.4). For multiplex immunoassaying, coronal sections were incubated with several primary antibodies for 12 h at 4°C. The primary antibodies used in this study were monoclonal rat anti-BrdU IgG (1:10; Oxford Biotechnology, UK), monoclonal mouse anti-GAD67 (GAD) IgG (1:200; Millipore, USA), monoclonal mouse anti-glutamate transporter (Glu) IgG (1:200; Millipore, USA), and goat polyclonal anti-c-Fos antibody (1:500; SantaCluz, Santa Cruz, USA). To identify the localization of BrdU-immunopositive (BrdU+) cells co-labeled with GAD, Glu, and c-Fos, Alexa Fluor 633 anti-rat IgG with Alexa Fluor 488 anti-mouse IgG and Alexa Fluor 488 anti-mouse IgG (1:500; Molecular Probes, OR, USA) as secondary antibodies. After staining, sections were mounted on glass slides and mounted with 80% glycerol.

### Cell counting

A confocal microscope (FV-1000D; Olympus, Japan) and imaging software (Fluoview; Olympus, Japan) were used to visualize all sections under 20× or 40× magnifications. An Alexa 633 filter was used to observe BrdU+ cells, and an Alexa 488 filter was used to detect the other co-labeled cells. For the hypothalamic area, brain sections (between −0.26 and −4.80 mm from the bregma) were obtained according to the Paxinos and Watson atlas [26]. Individual BrdU+ cells stained with GAD, Glu, or c-Fos were also counted. Immunopositive cells were counted in 12 sections per animal, as described previously [23, 25]. Because the immuno-labeled cells were counted at one-sixth interval sections, the possibility of counting split cells on different sections was minimized to <10%, according to the equation of Abercrombie.

### Western blot analysis

Hypothalamic sections were extracted with lysis buffer composed of 1 mM EDTA, 1% SDS, 1x Complete protease inhibitor cocktail (Roche Diagnostics, Schweiz), and 10 mM Tris-HCl (pH 7.5). The lysate was sonicated and centrifuged at 14,000 rpm for 20 min at 4°C to obtain the supernatant as the cell extract. The lysates were then analyzed by Western blotting as described previously [27, 28]. Briefly, lysates were separated on 10–12.5% SDS-PAGE and transferred onto PVDF membranes (Immobilon-P, Millipore, USA). Membranes were incubated with polyclonal rabbit anti-Dcx antibody (1:1000, Cell Signaling) or polyclonal rabbit anti-PCNA antibody (1:1000, Cell Signaling, MA, USA). HRP-conjugated anti-rabbit IgG (1:2000, Cell Signaling, MA, USA) were used as the secondary antibody. Immunoblots were incubated with the ECL detection kit (Amersham ECL Prime, GE Healthcare, UK) and visualized with an image analyzer (LAS-4000, FUJI FILM, Japan). Membranes were then stripped and reprobed with monoclonal rabbit anti-β-actin antibody as a loading control (1:2000; Cell Signaling, MA, USA).

### Reverse transcription-polymerase chain reaction analysis

Total RNA from hypothalamic sections was purified and reverse transcribed using a reverse transcription kit (TAKARA, Japan). To evaluate the mRNA expression profiles of brain derived neurotorophic factor (BDNF) and β-actin of the hypothalamus, reverse transcription-polymerase chain reaction (RT-PCR) was performed. mRNA levels were obtained using GoTaq (Promega, WI, USA) and the following primers: 5’-ggtcacagtcctggagaaag-3’ and 5’-gcttatccttatgaaccgcc-3’ for BDNF, and 5’-atggtgggtatgggtcagaag-3’ and 5’-ctggggtgttgaaggtctcaa-3’ for β-actin.

### Enzyme-linked immunosorbent assay (ELISA)

Hypothalamus sections were homogenized with tris-buffer (pH 7.4) and centrifuged at 800 ×*g* for 15 min at 4°C to remove tissue debris. Protein assays were performed using Pierce BCA Protein Assay Kit (Thermo Fisher Scientific, MA, USA) to determine protein concentration. Equal amount of protein were analyzed with the BDNF Emax^®^ ImmunoAssay System (Promega, WI, USA) according to the manufacturer’s protocol. Absorbance at 450 nm was measured by a plate reader (DTX880, Beckman Coulter, CA USA), and BDNF concentrations were calculated using SoftMax pro software (Molecular Devices, LLC).

### Intracerebral ventricle infusion of AraC

To eliminate hypothalamic progenitor cell proliferation, cytosine arabinoside (AraC, Sigma, St Louis, MO, USA) was chronically infused into the intracerebral ventricle by using an osmotic pump (MODEL2006, Alzet, CA, USA). Rats were anesthetized, the skull was exposed and a small hole (bregma; 0.8 mm posterior and 1.4 mm lateral) was drilled according to the atlas of Paxinos and Watson using a stereotaxic frame (Narishige, Japan). The osmotic pump contained 235 μL of AraC dissolved in saline (1 μg/μL) and was infused at an infusion rate of 0.15 μL/h over 42 days (AraC rats). This dose of AraC effectively blocked cell proliferation in the hypothalamus without causing overt adverse effects on health [11, 14]. Saline-infused rats were used as a vehicle group (Veh rats). Non-operated rats (Naive rats) and sham-operated rats (Sham rats) were also prepared. All rats were allowed to recover from surgery for 10 days. After the recovery period, AraC and Veh rats were subjected to a constant T_a_ of 32.0 ± 0.2°C for 40 days (AraC+HA and Veh+HA), while respective CN rats (AraC+CN and Veh+CN) were kept at a T_a_ of 24.0 ± 0.1°C. After 40-day of heat exposure, rats were subjected to a heat tolerance test and the behavioral thermoregulation of rats was investigated. Subsequent to this, brains were removed and used for immunohistochemical analysis.

### Estimation of behavioral thermoregulation

Behavioral thermoregulation was estimated by their selected ambient temperatures (T_s_) as described previously [27, 29]. Briefly, a long wire mesh cage (200 × 12 × 12 cm) located in an outer temperature gradient box was constructed from 0.78-cm-thick aluminum with dimensions of 210 × 15 × 15 cm. Eighteen position sensors activated by an infrared light source (PZ-41, KEYENCE, Japan) were located along the length of the temperature gradient at approximately 10-cm intervals. The ends of the outer gradient box were maintained at 15°C and 37°C with water perfusion devices, which resulted in air temperatures inside the box ranging from approximately 17°C to 36°C. AM receivers (RLA3000, Data Sciences, St Paul, MN, USA) were placed on the temperature gradient box. Food was placed on the floor of the internal cage and water was provided through four holes made in the ceiling of the temperature gradient box. Thus, the rats could obtain food and water at their preferred ambient temperature. Light control was maintained throughout the experiments. Location of rats in the thermal gradient was detected by the position sensors and T_s_ was estimated by the calibration curve of air temperature as a function of location. Measurements started at 16:00 hours and continued for 3 days. T_ab_ and T_s_ data were sampled with a computer logging system. The spontaneous activity levels of rats were estimated according to the number of times that rats crossed the infrared beam emitted from the position sensors in one minute.

### Heat tolerance test

The heat tolerance test was carried out as described previously [25, 27]. Briefly, rats were subjected to a 180-min thermal gradient that was increased by 0.7–0.8°C every 10 min from 24–36°C. When rats were subjected to the heat tolerance test, the abdominal temperature (T_ab_) of rats in each group was measured using the telemetry system (Dataquest; Data Sciences International, St Paul, MN, USA). Food and water were removed during the test. After the test, brains were removed and analyzed immunohistochemically.

### Data quantification and statistical analysis

Results are presented as the mean ± S.E.M. Statistical analyses were performed with SPSS software (IBM, version 22). The parameters obtained from telemetry, T_s_, and cell counts were analyzed using analysis of variance with Tukey’s post hoc test. Quantitative data from Western blotting, ELISA, and RT-PCR were analyzed by two-tailed t-test. A p value < 0.05 was considered to be statistically significant.

## Results

### Proliferation of newborn cells in the hypothalamus

**Fig 1:**
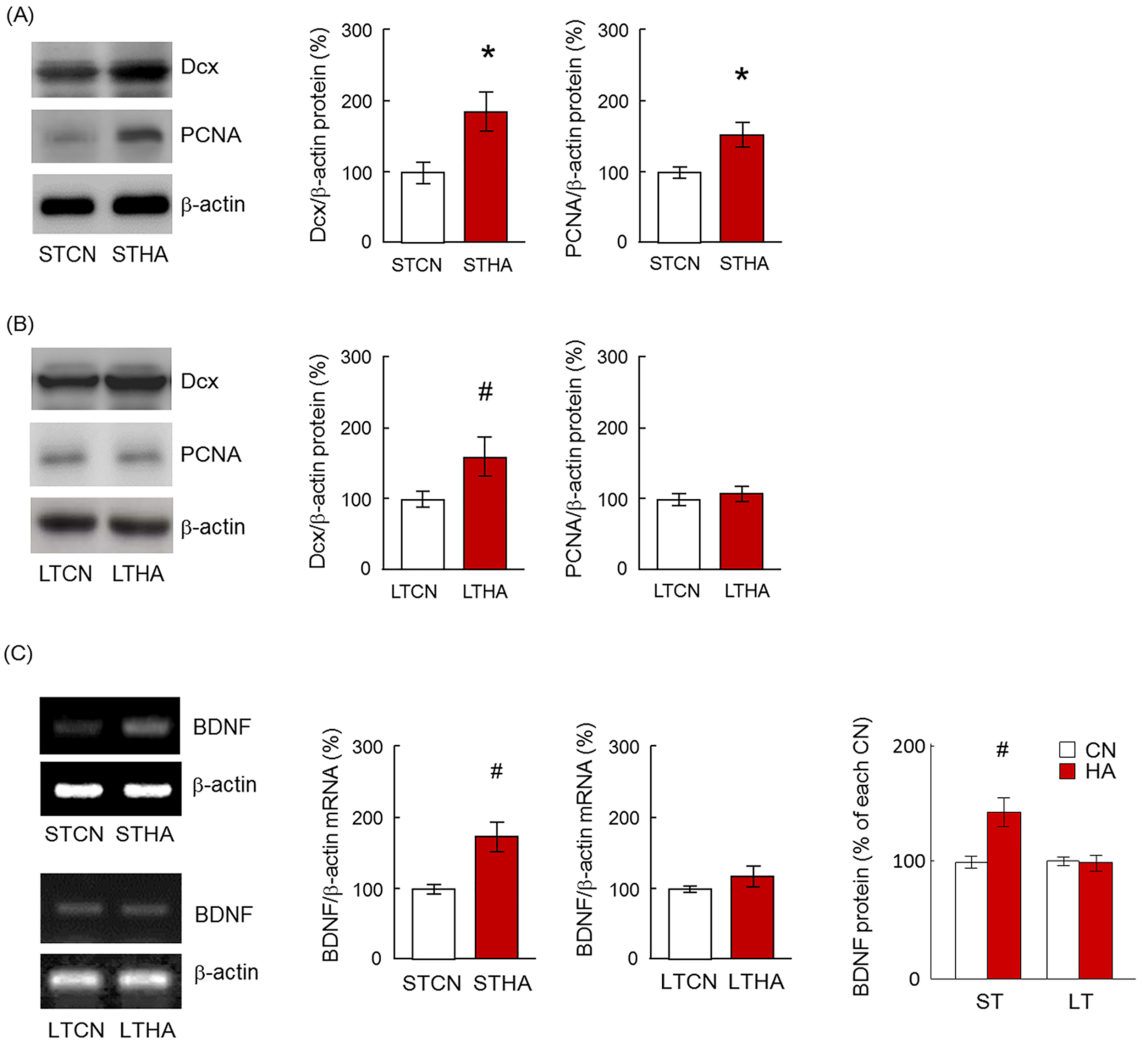
(A) Protein expression levels of Dcx and PCNA in the hypothalamus of STCN and STHA rats. (B) Protein expression levels of Dcx and PCNA in the hypothalamus of LTCN and LTHA rats. (C) BDNF mRNA and protein expression in the hypothalamus. Values are the means ± SEM* significant difference between CN and HA groups (* p < 0.01, # p < 0.05, n = 6 in each group).

Previously, we reported that the number of BrdU+ cells in the hypothalamus was significantly increased in 6- to 53-day heat-exposed rats [23]. In this study, we reconfirmed anew that the number of BrdU+ cells in the hypothalamus of STHA and LTHA rats were significantly increased compared with those of respective control rats (data not shown). To ascertain that cell proliferation definitely occurs in the hypothalamus of HA rats, the protein expression levels of PCNA and Dcx were analyzed (Fig 1A and 1B). PCNA is a homotrimer and achieves its processivity by encircling DNA, wherein it acts as a scaffold to recruit proteins involved in DNA replication, DNA repair, cell cycle regulation, and chromatin assembly [30]. Dcx is a microtubule-associated protein expressed by neuronal precursor cells and immature neurons. Neuronal precursor cells begin to express Dcx while actively dividing, and their neuronal daughter cells continue to express Dcx for 2–3 weeks as the cells mature into neurons [31]. The protein expression levels of both PCNA and Dcx are kept high during the development of newborn neurons within certain areas in the adult mammalian brain. The protein expression levels of Dcx and PCNA in the hypothalamus of the STHA group were significantly increased compared with those of the STCN group (Fig 1A, Dcx; p < 0.01, PCNA; p < 0.01). In the LTHA group, Dcx protein expression level in the hypothalamus was significantly higher than that of the LTCN group (p < 0.05), whereas PCNA protein expression was not altered (Fig 1B, p = 0.62). BDNF mRNA and protein expression levels in the hypothalamus were also investigated (Fig 1C) because by enhancing cell survival, this neurotorophic factor can promote protective pathways and inhibit damaging pathways that contribute to neurogenic response in the brain [32]. mRNA and protein expression of BDNF in the hypothalamus of the STHA group increased significantly compared to that of the STCN group (Fig 1C, p < 0.05). However, both mRNA and protein levels of BDNF in the hypothalamus of the LTHA group were not altered (p = 0.86).

### GAD67 and glutamate transporter expression in hypothalamic newborn cells

**Fig 2:**
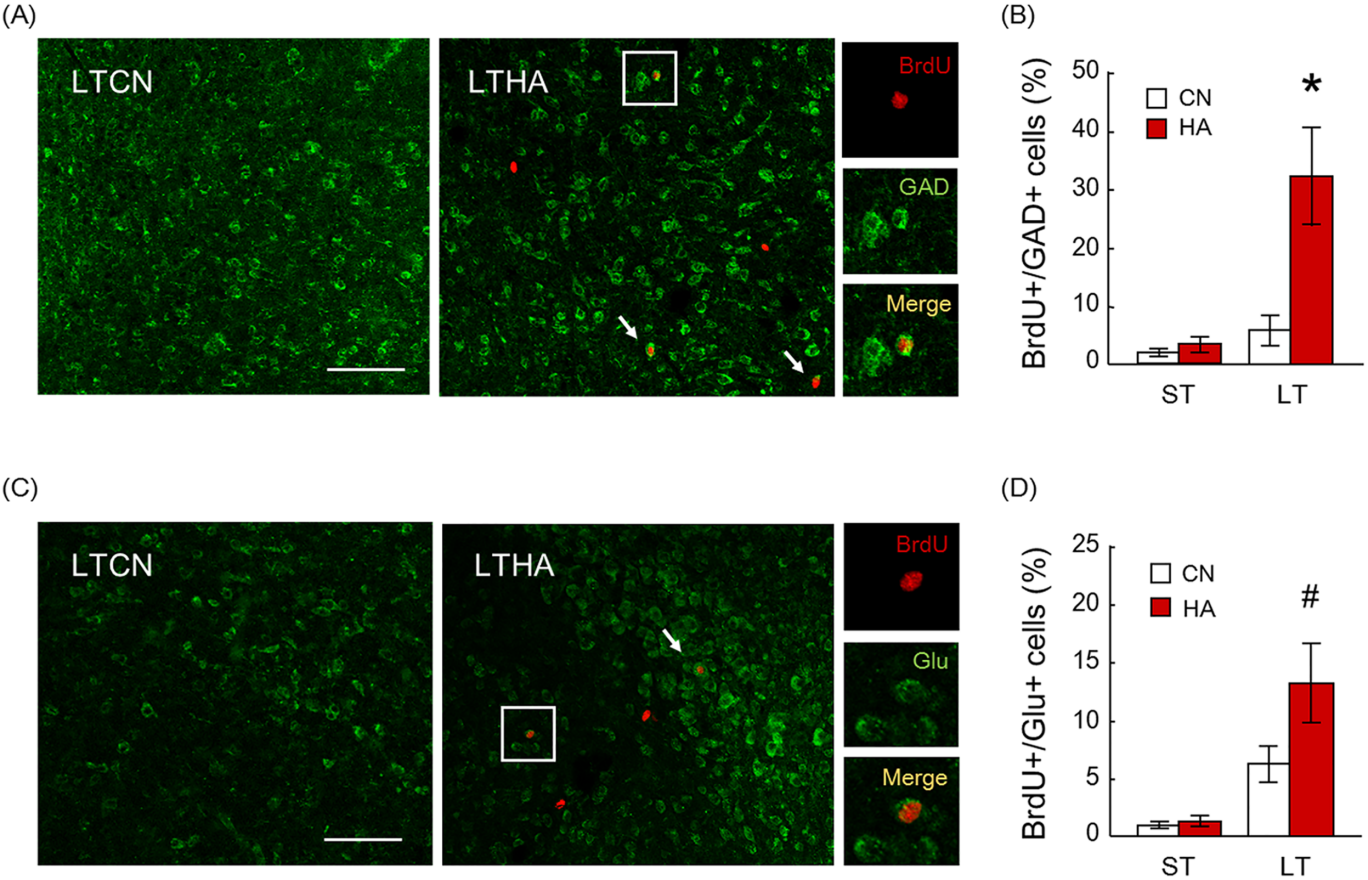
(A) BrdU/GAD immunostaining. Arrows denote BrdU/GAD immunopositive (BrdU+/GAD+) cells. Bar; 100 μm. Right panels show magnified views of boxed regions from LTHA rats. (B) The percentage of BrdU+/GAD+ cells to BrdU+ cells in the hypothalamus was significantly increased with 40-day heat exposure. Values are the means ± SEM (n = 6 in each group). * significant difference between LTCN and LTHA groups (p < 0.01). (C) BrdU/Glu immunostaining. Arrows denote BrdU/Glu immunopositive (BrdU+/Glu+) cells. Bar; 100 μm. Right panels show magnified views of boxed regions from LTHA rats. (D) The percentage of BrdU+/Glu+ cells to BrdU+ cells in the hypothalamus was significantly increased with 40-day heat exposure. Values are the means ± SEM (n = 6 in each group). # significant difference between LTCN and LTHA groups (p < 0.05).

Hypothalamic sections were immunolabeled with the anti-BrdU antibody together with the anti-GAD67 antibody, which labels the cytoplasm of GABAergic neurons (Fig 2A). BrdU and GAD67 double-labeled (BrdU+/GAD+) cells were rarely detected in the hypothalamus of STCN, STHA, and LTCN groups. The percentages of BrdU+/GAD+ cells to total BrdU+ cells in STCN, STHA, and LTCN groups were 2.7%, 3.6% and 6.2%, respectively (Fig 2B). While in the LTHA group, many BrdU+/GAD+ cells were found in the hypothalamic parenchyma (Fig 2A). In LTHA group, approximately 32.6% of BrdU+ cells in the preoptic area (POA) of the anterior hypothalamus were stained by GAD67 (Fig 2B). The percentage of BrdU+/GAD+ cells to BrdU+ cells in the hypothalamic area increased significantly in the LTHA group compared with to the LTCN group (p < 0.05, Fig 2B). Brain sections were immunolabeled with the anti-BrdU antibody together with the anti-glutamate transporter antibody, which labels the cytoplasm of glutamatergic neurons (Fig 2C). BrdU and glutamate transporter double-labeled (BrdU+/Glu+) cells were visualized and the percentage of BrdU+/Glu+ cells to BrdU+ cells in the hypothalamic area was counted. In LTHA rats, 15.2% of BrdU+ cells were stained by Glu (Fig 2D). The percentage of BrdU+/Glu+ cells to BrdU+ cells in the hypothalamic area increased significantly in the LTHA group compared with the LTCN group (6.6%, p < 0.05) while very few BrdU+/Glu+ cells in STCN (1.5%) and STHA groups (1.9%) were detected (Fig 2D).

### c-Fos expression in hypothalamic newborn cells

**Fig 3:**
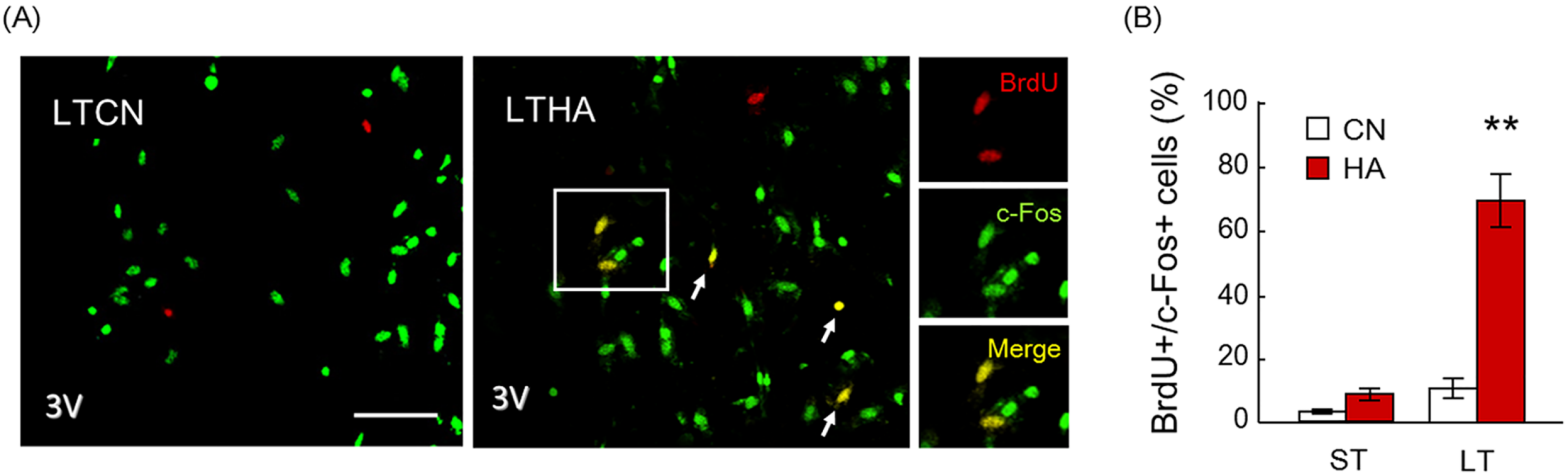
(A) BrdU/c-Fos immunostaining. Arrows denote BrdU/c-Fos immune positive (BrdU+/c-Fos+) cells in the hypothalamus. 3V; third ventricle. Bar; 100 μm. Right panels show magnified views of boxed regions from LTHA rats. (B) The percentage of BrdU+/c-Fos+ cells in the hypothalamus was significantly increased with 40-day heat exposure. Values are the means ± SEM (n = 4 in each group). ** significant difference between LTCN and LTHA groups (p < 0.001).

Candidate populations of functional newborn cells in the POA were identified as BrdU+ cells, which express the c-Fos protein, a proto-oncogene involved in diverse cellular functions following in response to noxious stimulation, tissue injury, and/or thermal stimuli [33]. Hypothalamic sections were immunolabeled with the anti-BrdU antibody and c-Fos antibody (Fig 3A). BrdU and c-Fos double-labeled cells (BrdU+/c-Fos+ cells) were counted and the percentage of BrdU+/c-Fos+ cells to BrdU+ cells in the hypothalamus was calculated. In STCN and STHA groups, very few BrdU+/c-Fos+ cells were detected in the hypothalamus. In addition, BrdU+/c-Fos+ cells in the LTCN group were infrequently located. The percentage of BrdU+/c-Fos+ cells to BrdU+ cells in the hypothalamus was only 10.4%. However, numerous BrdU+/c-Fos+ cells were detected in the LTHA group (Fig 3A). Indeed, 63.2% of BrdU+ cells in the hypothalamus were immunolabeled with the c-Fos antibody (Fig 3B, p < 0.0001).

### T_ab_ and the locomotor activity of AraC-infused rats

The mean T_ab_ and locomotor activity in the light and dark phases of day were measured with the telemetry system and are summarized in Tables 1 and 2. Before starting heat exposure (Before), T_ab_ levels and the locomotor activity of all rats at the T_a_ of 24°C did not differ (Tables 1 and 2). During heat exposure (During), the T_ab_ levels of HA+Veh increased significantly both in the light (Table 1, p < 0.0001, CN+Veh vs. HA+Veh) and dark phases (Table 1, p < 0.0001, CN+Veh vs. HA+Veh) in comparison with that of CN+Veh. AraC administration, however, did not affect T_ab_ levels in rats (p = 0.56, HA+Veh vs. HA+AraC). Locomotor activity during heat exposure in HA+Veh decreased significantly both in the light (Table 1, p < 0.0001, CN+Veh vs. HA+Veh) and dark phases (Table 2, p < 0.0001, CN+Veh vs. HA+Veh). However, AraC did not change the locomotor activity of both light (Table 2, p = 0.7, HA+Veh vs. HA+AraC) and dark phases (Table 2, p = 0.63, HA+Veh vs. HA+AraC).

**Table 1:** The mean T_ab_ values during the daily light and dark phases.

	Before		During	
Group	Light phase	Dark phase	Light phase	Dark phase
CN+Veh	37.17 ± 0.05	37.82 ± 0.05	37.20 ± 0.02	37.80 ± 0.06
CN+AraC	37.16 ± 0.07	37.81 ± 0.07	37.24 ± 0.05	37.76 ± 0.08
HE+Veh	37.15 ± 0.08	37.82 ± 0.06	37.72 ± 0.04*	38.72 ± 0.13*
HE+AraC	37.16 ± 0.07	37.82 ± 0.07	37.79 ± 0.07*	38.62 ± 0.11*

Before, a day before starting the heat exposure period; During, the last day of the heat exposure period; Day, days after starting the heat exposure period; CN+Veh, vehicle-infused control rats; CN+AraC, AraC-infused control rats; HA+Veh, vehicle-infused heat-acclimated rats; HA+AraC, AraC-infused heat-acclimated rats. Values are the means ± SEM (n = 4 in each group).

* significant difference between Veh-infused and AraC-infused groups (p < 0.05).

**Table 2:** The mean locomotor activity during the daily light and dark phases.

	Before		During	
Group	Light phase	Dark phase	Light phase	Dark phase
CN+Veh	1.065 ± 0.46	3.725 ± 1.05	1.625 ± 0.82	3.280 ± 0.54
CN+AraC	0.966 ± 0.57	3.256 ± 0.77	1.223 ± 0.45	3.576 ± 0.72
HE+Veh	1.015 ± 0.42	3.169 ± 1.06	0.620 ± 0.74*	1.072 ± 0.10*
HE+AraC	1.216 ± 0.54	3.458 ± 1.17	0.579 ± 0.67*	0.962 ± 0.11*

Before, a day before starting the heat exposure period; During, the last day of the heat exposure period; Day, days after starting the heat exposure period; CN+Veh, vehicle-infused control rats; CN+AraC, AraC-infused control rats; HA+Veh, vehicle-infused heat-acclimated rats; HA+AraC, AraC-infused heat-acclimated rats. Values are the means ± SEM (n = 4 in each group).

* significant difference between Veh-infused and AraC-infused groups (p < 0.05).

### BrdU+ cells in the hypothalamus of AraC-infused rats

**Fig 4:**
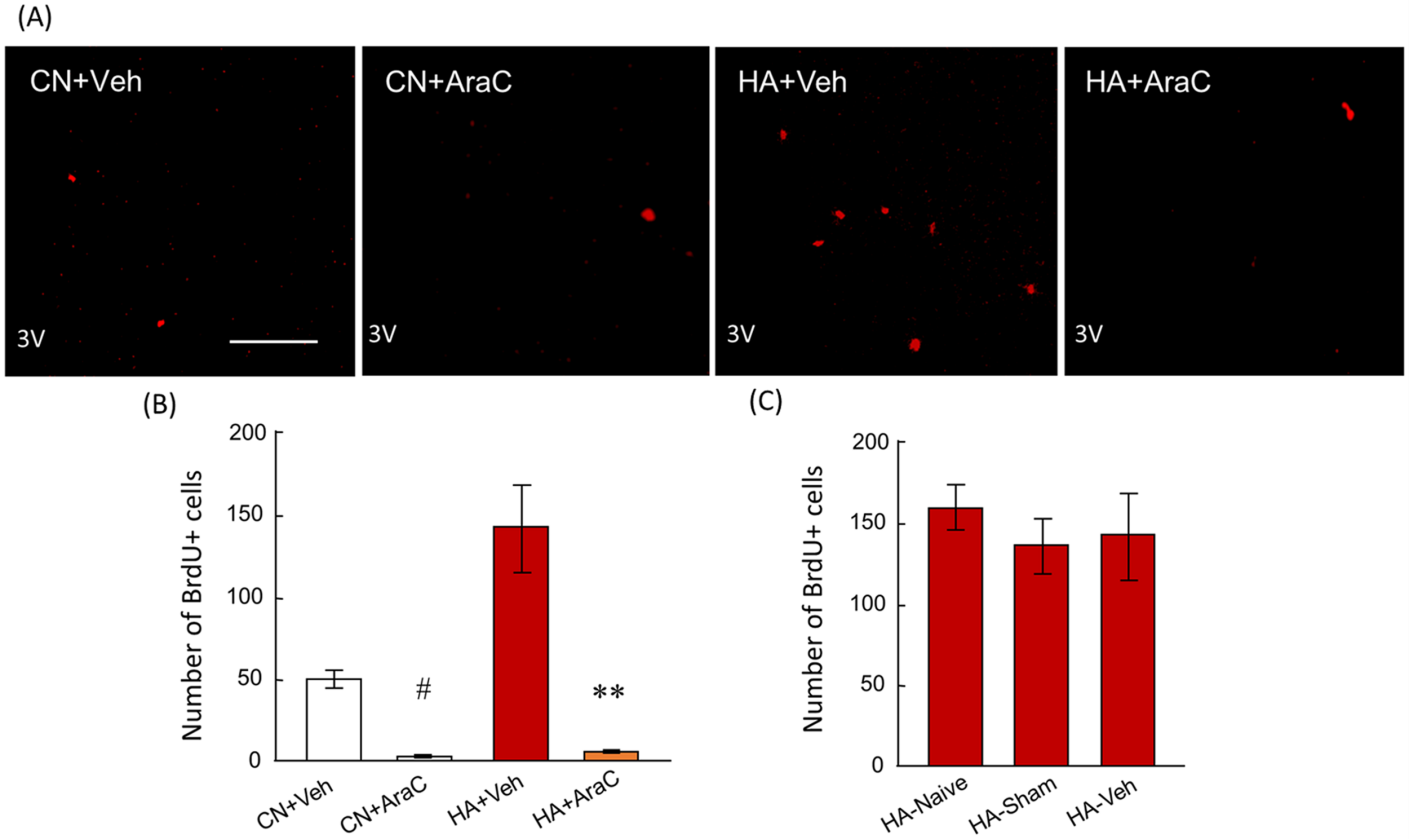
(A) BrdU immunostaining of CN+Veh, CN+AraC, HA+Veh, and HA+AraC groups. Bar; 100 μm. (B) The number of BrdU+ cells in the hypothalamus of CN+Veh (white bar), CN+AraC (gray bar), HA+Veh (red bar), and HA+AraC (beige bar) rats. (C) The number of BrdU+ cells in the hypothalamus of HA-naive, HA-Sham, and HA-Veh rats. Sham-operation and vehicle infusion did not change the number of BrdU+ cells in the hypothalamus. Values are the means ± SEM (n = 4 in each group). #, significant difference between CN+Veh and CN+AraC groups (p < 0.01). ** significant difference between HA+Veh and HA+AraC groups (p < 0.001).

In both CN+Veh and HA+Veh groups, BrdU+ cells were detectable in the hypothalamus (Fig 4A). The number of BrdU+ cells in the HA+Veh group was significantly higher than that of the CN+Veh group (Fig 4B, p < 0.01). However, very few BrdU+ cells were detected in the hypothalamus in CN+AraC and HA+AraC rats (Fig 4A). The number of BrdU+ cells in CN+AraC and HA+AraC groups was significantly lower than that of CN+Veh and HA+Veh groups, respectively (Fig 4B, p < 0.001; CN+Veh versus CN+AraC, p < 0.0001; HA+Veh versus HA+AraC). Additionally, the number of BrdU+ cells in heat-acclimated naive rats (HA-naive), sham-operated HA rats (HA-sham), and vehicle-infused HA rats (HA-Veh) were counted. No change in the number of BrdU+ cells in the hypothalamus of HA-sham and HA+Veh rats with HA-naive rats were recorded (Fig 4C).

### Heat tolerance of AraC-infused rats

**Fig 5:**
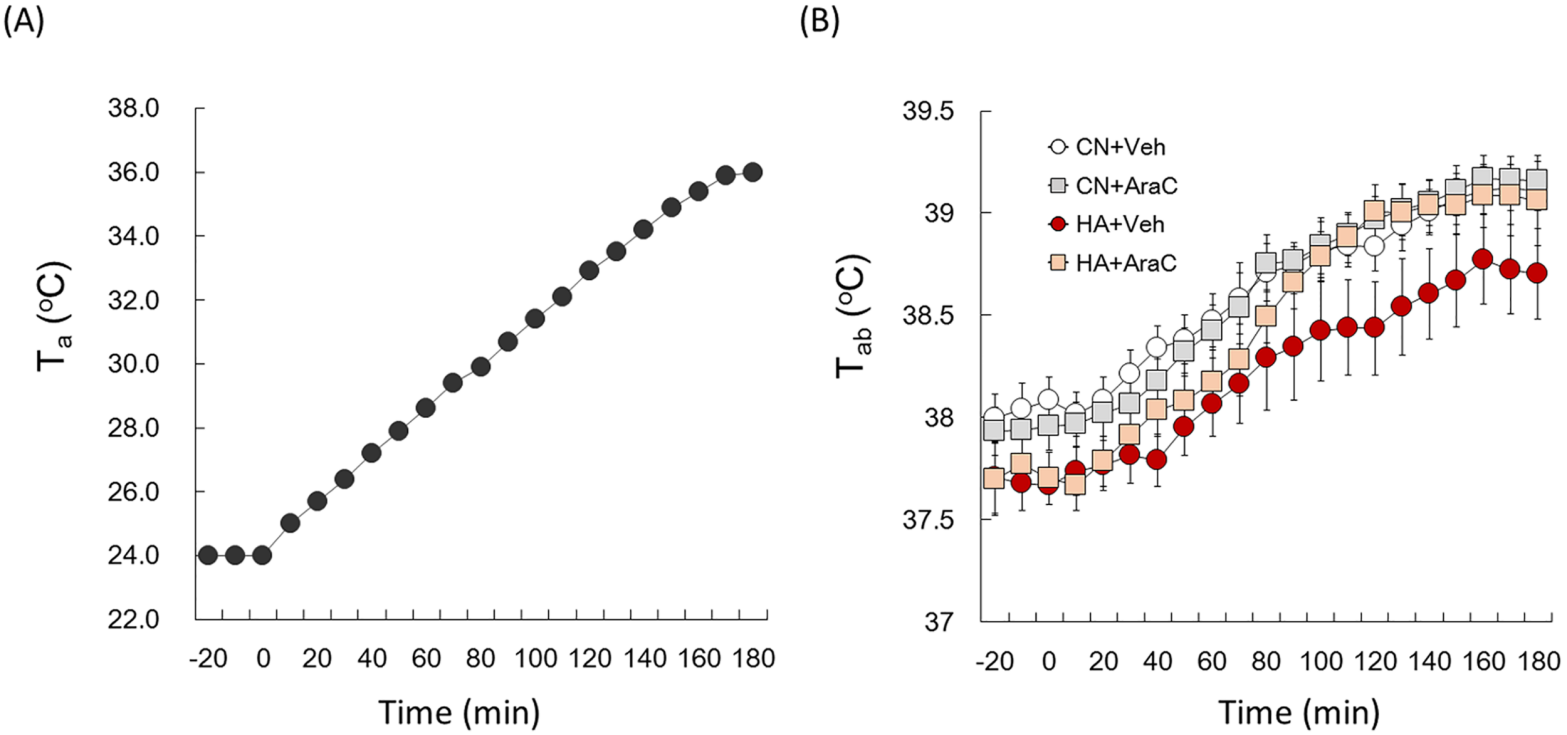
(A) The T_a_ of the chamber in the heat tolerance test. Rats were subjected to a 180-min thermal load. The T_a_ was increased by 0.7–0.8°C every 10 min from 24°C to 36°C. (B) T_ab_ responses to heat in CN+Veh (white circle), CN+AraC (gray square), HA+Veh (red circle), and HA+AraC (beige square) rats. Values are the means ± SEM (n = 4 in each group).

During the heat tolerance test, the T_a_ was raised from 24°C to 36°C as shown in Fig 5A. T_ab_ values of all the groups during the heat tolerance test are shown in Fig 5B, where T_ab_ gradually increased with time. AraC administration into the intracerebral ventricle of CN rats did not alter the T_ab_ rise during the heat tolerance test (Fig 5B, F_1, 19_ = 11.8, p = 0.69). T_ab_ values of the HA+Veh group were lower over time compared with those of the CN+Veh group (Fig 5B, F_1, 19_ = 21.8, p < 0.001). Initial levels of T_ab_ (during -20 to 0 min) of both the HA+Veh and HA+AraC groups were significantly lower than those of the CN+Veh and CN+AraC groups (Fig 5B), respectively. However, T_ab_ values of the HA+AraC group during 110 min to 180 min of the test were significantly higher than those of the HA+Veh group (Fig 5B, F_1, 19_ = 11.8, p < 0.05).

### T_s_ of AraC-infused LTHA rats

**Fig 6:**
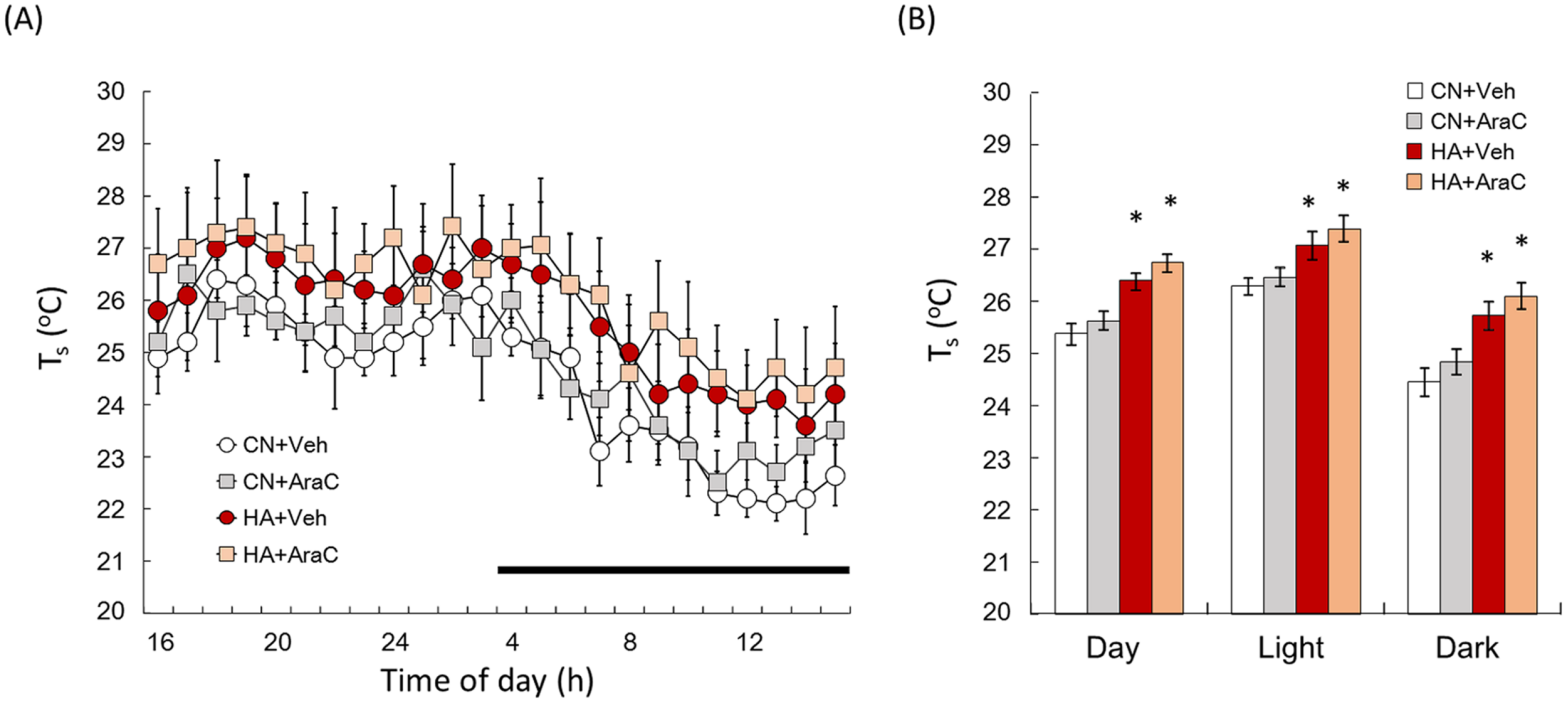
(A) T_s_ values of Day in CN+Veh (white circle), CN+AraC (gray square), HA+Veh (red circle), and HA+AraC (beige square) rats. Dark bars above the abscissa indicate the dark phase of the day. (B) Mean T_s_ in Day, light and dark phases. Forty-day heat exposure increased in the value of T_s_. However, AraC administration did not affect heat exposure-induced changes of T_s_ in HA rats. Values are the means ± SEM (n = 4 in each group). *, significant difference between CN+Veh and HA+Veh or CN+AraC and HA+AraC (p < 0.05) groups.

T_s_ values were measured by the thermal gradient chamber. There were clear day-night variations in T_s_ values in all of the groups (Fig 6B). T_s_ values in the HA+Veh group were higher than those of the CN+Veh group (Fig 6A). The mean T_*s*_ of the HA+Veh group in the day (Day), light phase (Light), and dark phase (Dark) was significantly higher than that of the CN+Veh group (Fig 6B, Day; p < 0.01, Light; p < 0.01, Dark; p < 0.01). The T_s_ value and Mean T_s_ of the CN+AraC group during day, light, and dark was almost the same as the CN+Veh group. In addition, the T_s_ value and mean T_s_ during day, light, and dark did not differ between HA+Veh and HA+AraC groups (Fig 6A and 6B).

## Discussion

In this study, we examined the expression levels of PCNA, a cell proliferation marker, and Dcx, a neural differentiation marker, in the hypothalamus of HA rats. In the STHA group, expression levels of both PCNA and Dcx in the hypothalamus increased in compared with those of the STCN group. In the LTHA group, the expression level of PCNA did not enhance, whereas the expression level of Dcx was significantly higher than that of the LTCN group. Previously, we have reported that progenitor cell proliferation and neural differentiation in the hypothalamus is enhanced in HA rats [23]. The number of BrdU+ cells in the hypothalamic area of HA rats was significantly greater than that of CN rats. The proliferation started within the first 5 days of the heat exposure period and persisted for at least the following 20 days. A large number of BrdU+ cells in the STHA rats were detected in the ependymal layer of the third ventricle, where progenitor cells are shown to exist [10]. In contrast, BrdU+ cells of LTHA rats were broadly expressed in the parenchyma of the hypothalamus. Taken together with BrdU immunostaining, results suggest that heat exposure initiated the proliferation of the progenitor cells in the hypothalamus within a few days after heat exposure, and progenitor cell migration and neuronal cell differentiation were facilitated thereafter. Our previous study indicated that the number of hypothalamic BrdU+ cells co-labeled with neuronal nuclei (NeuN), a mature neuron marker, in heat-acclimated rats increased drastically with 30–40 days of heat exposure, while BrdU+ cells co-labeled with glial markers were detected rarely [23–25]. These findings indicate that most of the hypothalamic newborn cells induced by heat exposure differentiate into neurons. Because delineating the specific neural cell types involved in thermoregulation is a key step toward understanding these critical neural circuits, we investigated the neural phenotype of hypothalamic BrdU+ cells. Although various types of nerve cells exist in the POA, GABAergic neurons are known to participate in autonomic thermoregulation in mammals [4, 6]. For example, GABAergic inhibitory inputs from the POA to the DMH and rostral medullary raphe region (rMR) suppress thermogenesis in brown adipose tissue (BAT) and vasoconstriction. Cold-induced inhibition of these signals promotes BAT thermogenesis [4]. From this point of view, we analyzed the expression of the GABAergic neuron marker of hypothalamic BrdU+ cells. In the POA, approximately 30% of BrdU+ cells are immunolabeled with GAD. Our preliminary analysis using Alexa-Fluor488-conjugated cholera toxin b subunit (CTb), a retrograde neuron tracer, indicated that a portion of BrdU+ cells in the POA is stained with CTb, which was injected into the DMH (data not shown). It may be necessary to analyze projections of the POA to the rMR in newborn neurons of LTHA rats in future experiments.

BDNF was transcriptionally upregulated in the hypothalamus of STHA rats, but this was not the case in LTHA rats (Fig 1C). Umschweif et al reported that the expression level of BDNF is transiently enhanced by heat exposure via the upregulation of hypoxia-inducible factor 1α (HIF-1α) [34]. We had previously proven that heat exposure enhances HIF-1α-mediated pathway in NIH3T3 mouse fibroblast cells [35, 36]. BDNF, a classic neurotrophic factor, plays a significant role in neurogenesis and synaptogenesis. BDNF can also promote neural stem cells (NSCs) proliferation through Akt activation and PTEN inactivation [32, 37]. There have been many *in vivo* studies demonstrating BDNF as a strong promoter of neuronal differentiation [38–40]. Infusion of BDNF into the lateral ventricle doubled the population of newborn neurons in several brain regions of the adult rat [41]. Although the exact role of the transient upregulation of BDNF in HA rats is unknown, heat exposure may affect neural progenitor proliferation and nerve growth and cause remodeling of thermoregulatory networks in the hypothalamus via the functions of neurotrophic factors.

The POA is known to receive input from cold- and heat-sensitive neurons, and c-Fos in the POA is upregulated by thermal stimuli. It is now widely accepted that c-Fos+ cells in the POA play a key role in autonomic thermoregulation in heat environments [33]. Dysfunction of POA in rodents shows a reduced ability of autonomic thermoregulation; however, they can conduct behavioral thermoregulation [42, 43]. To identify the activity of hypothalamic BrdU+ cells, we used c-Fos staining to identify newborn cells that responded to thermal stimulation. Our immunohistochemical analysis revealed that approximately 67% of BrdU+ cells in the POA of LTHA rats are co-labeled with c-Fos, while BrdU+/c-Fos+ cells were minimally detected in STHA rats (Fig 3). These results indicate that BrdU+/c-Fos+ cells in the POA may mediate autonomic thermoregulation in LTHA rats.

We next determined whether inhibiting these newborn neurons in the hypothalamus was sufficient to drive heat acclimation. Using the mitosis blocker, AraC, the ability of heat tolerance was impaired in HA+AraC rats (Fig 4B). It has been reported that AraC, this mitotic blocker, can also act as a cytotoxin, triggering the apoptotic degradation of postmitotic neurons in culture [44]. To address this potential concern, AraC–treated brains were stained with the sensitive cell death marker Fluoro-Jade. No signs of cell degeneration throughout the brain parenchyma after AraC treatment were observed (data not shown). AraC injection did not change T_ab_ (Table 1) or the locomotor activity (Table 2) of rats during light and dark phases. These results suggest that the improvement of acquired heat tolerance in HA rats depends on neural cell proliferation in the hypothalamus. We further measured T_s_ variation in AraC-infused rats using the thermal gradient system. The thermal gradient system is one of the ideal methods to study long-term changes in behavioral thermoregulation because a rat can simply select the optimal T_a_ to control its core body temperature by simply moving to a different location [29]. Furthermore, animals have a mechanism to optimize their energy expenditure for core temperature regulation by selecting the environment that corresponds to the lowest metabolic oxygen demand. Therefore, T_s_ measured by the thermal gradient system was used as one of the markers for behavioral thermoregulation. Although the 40-day heat exposure significantly enhanced T_s_ values in both light and dark phases, chronic infusion of AraC into the lateral ventricle did not change the T_s_ variation in rats (Fig 6). This result suggests that heat-induced newborn cells in POA and other brain regions do not contribute to changes in the behavioral thermoregulatory functions in HA rats. Though the central mechanism of behavioral thermoregulation has not been fully identified, the neural circuit from skin thermoreceptors to the cerebral cortex for somatic thermal sensation has been thoroughly studied [45, 46]. Neurons responding to non-noxious thermal stimuli applied to the skin are located in the lamina I of the spinal cord, and signals from these neurons primarily reach the posterior part of the ventral medial nucleus in the thalamus. Previous studies have shown that these signals reach several areas in the cerebral cortex, such as the insula, primary and secondary somatosensory, orbitofrontal, and cingulate cortex [47–49]. Although the specific roles of the cerebral cortex for behavioral thermoregulation are unclear, the cerebral cortex may be attributable to behavioral thermoregulation. Our previous study proved that heat exposure did not increase the number of BrdU+ cells in the subventricular zone of cortex where neural progenitor cells are abundantly exist [23]. Newly generated neurons in the hypothalamus may be involved in autonomic thermoregulation, such as vasodilatation and the suppression of BAT thermogenesis, but probably do not participate in behavioral thermoregulation in HA rats. AraC, however, affects proliferation of not only neuronal precursors but also other proliferative cells, e.g., microglia, in the hypothalamus. Microglia cells appear to play an important role during normal function of the mature nervous system. In response to injury, ischemia, and inflammatory stimuli, microglia cells assume an activated phenotype associated with proliferation, migration to the site of injury, phagocytosis of cellular debris, and elaboration of both neurotoxic and neurotrophic factors [50]. Though exact relationship between microglia and heat acclimation is unknown, the proliferation of microglia in the hypothalamus of HA rats should be examined in the future.

There are many studies that demonstrate the stress, including severe heat stress, attenuating generation of new cells of both neural and not-neural origins [51–53]. However, several studies report proliferative effects by mild heat in numerous cell types [54–56]. Our preliminary *in vitro* study using NSCs in culture revealed that moderate heat exposure promotes NSCs proliferation, whereas intense heat exposure induces apoptosis-like cell death (data not shown). This suggests that the promotion and/or suppression of progenitor cell proliferation in the brain may be dependent on the level of heat stress. Changes in the rate of neuronal progenitor cell proliferation of the hypothalamus and acquired heat tolerance of HA rats under different T_a_ conditions should be studied in the future.

In conclusion, heat exposure generated GABAergic and/or glutamatergic neurons in POA and a part of the newborn cells in POA have responsiveness to heat stimuli. Inhibition of neural cell proliferation interfered with the acquired heat tolerance in rats without affecting the behavioral thermoregulation estimated by T_s_. Although the exact role of hypothalamic newborn neurons for autonomic thermoregulation are unknown, these observations suggest that heat exposure generates heat-sensitive functional neurons in POA that may have a certain relationship for establishing LTHA in rats.
